# Targeting nerve growth factor-mediated osteosarcoma metastasis: mechanistic insights and therapeutic opportunities using larotrectinib

**DOI:** 10.1038/s41419-024-06752-0

**Published:** 2024-05-30

**Authors:** Chun-Han Hou, Wei-Li Chen, Chih-Yang Lin

**Affiliations:** 1 Department of Orthopedic Surgery, National Taiwan University Hospital, No. 1, Jen-Ai Road, Taipei, 100 Taiwan, ROC; 2 Translational Medicine Center, Shin Kong Wu Ho-Su Memorial Hospital, Taipei, 111 Taiwan, ROC

**Keywords:** Bone cancer, Drug development, Cell invasion

## Abstract

Osteosarcoma (OS) therapy presents numerous challenges, due largely to a low survival rate following metastasis onset. Nerve growth factor (NGF) has been implicated in the metastasis and progression of various cancers; however, the mechanism by which NGF promotes metastasis in osteosarcoma has yet to be elucidated. This study investigated the influence of NGF on the migration and metastasis of osteosarcoma patients (88 cases) as well as the underlying molecular mechanisms, based on RNA-sequencing and gene expression data from a public database (TARGET-OS). In osteosarcoma patients, the expression of NGF was significantly higher than that of other growth factors. This observation was confirmed in bone tissue arrays from 91 osteosarcoma patients, in which the expression levels of NGF and matrix metallopeptidase-2 (MMP-2) protein were significantly higher than in normal bone, and strongly correlated with tumor stage. In summary, NGF is positively correlated with MMP-2 in human osteosarcoma tissue and NGF promotes osteosarcoma cell metastasis by upregulating MMP-2 expression. In cellular experiments using human osteosarcoma cells (143B and MG63), NGF upregulated MMP-2 expression and promoted wound healing, cell migration, and cell invasion. Pre-treatment with MEK and ERK inhibitors or siRNA attenuated the effects of NGF on cell migration and invasion. Stimulation with NGF was shown to promote phosphorylation along the MEK/ERK signaling pathway and decrease the expression of microRNA-92a-1-5p (miR-92a-1-5p). In in vivo experiments involving an orthotopic mouse model, the overexpression of NGF enhanced the effects of NGF on lung metastasis. Note that larotrectinib (a tropomyosin kinase receptor) strongly inhibited the effect of NGF on lung metastasis. In conclusion, it appears that NGF promotes MMP-2-dependent cell migration by inhibiting the effects of miR-92a-1-5p via the MEK/ERK signaling cascade. Larotrectinib emerged as a potential drug for the treatment of NGF-mediated metastasis in osteosarcoma.

## Introduction

Osteosarcoma (OS) is the most prevalent primary malignant tumor among bone tumors, primarily affecting children and adolescents. Males are more susceptibility to the disease, as indicated by a male-to-female ratio ranging from 1.5 to 2:1 [1]. OS is common to specific sites, including the distal femur (42%), proximal tibia (19%), and proximal humerus (10%) [2]. Recent advances in chemotherapy agents have significantly improved the five-year survival rate for non-metastatic osteosarcoma from 15% to 60% [3]. Nonetheless, the prognosis for patients with metastasis remains poor. Roughly 25% of osteosarcoma patients present with metastasis at the time of diagnosis, and their five-year overall survival rate is below 30% [4]. Conventional chemotherapeutic agents tend to be ineffective against metastatic osteosarcoma, due to its heightened malignancy and propensity for invasion. There is a pressing need to identify new pre-treatment prognostic markers and develop new targeted drugs as adjuvants for chemotherapy.

Using bibliometric and visual methods, Wu et al. presented a comprehensive overview of matrix metalloproteinases (MMPs) in the context of osteosarcoma. Their research addressed the mechanisms by which MMPs promote the invasion and metastasis of osteosarcoma as well as the potential of MMPs as active targets against osteosarcoma. MMPs are a class of zinc- and calcium-dependent proteases that play crucial roles in regulating the expression of cytokines in various cell processes, including inflammation, cell migration, angiogenesis, and apoptosis [5]. Many MMPs have been linked to the progression, invasion and metastasis of various cancers, including osteosarcoma, ovarian cancer, colorectal cancer, breast cancer, and non-small cell lung cancer [5–8]. MMP overexpression (specifically MMP-2, MMP-9, or MMP-13) is a promising indicator for prognosis and pulmonary metastasis in osteosarcoma [4, 6]. MMP-2 plays a key role in tumor motility and is overexpressed in oral carcinoma, glioblastoma, bladder cancer, bone metastasis, and sarcoma. As for MMPs in the context of osteosarcoma, future research is likely to focus on elucidating the underlying mechanisms, exploring their specific role in osteosarcoma lung metastasis, and investigating their potential as targets for cancer therapy.

MicroRNAs (miRNAs) are endogenous regulatory small non-coding RNAs that recognize complementary sequences in the 3’-untranslated region (UTR) of target genes, resulting in mRNA degradation, translation inhibition, or activation [9]. miRNAs are roughly 22 nucleotides in length. The function post-transcriptionally and play a crucial role in cell proliferation, differentiation, apoptosis, and ontogenesis as well as in modulating gene expression [10]. Numerous studies have explored the strong association between miRNAs and the development of osteosarcoma. For instance, miR-18b-5p, miR-331-3p, and miR-744-5p have shown tumor-suppressive functions involving the growth, proliferation, metastasis, and invasion of osteosarcoma cells [11–13]. Previous research has indicated that the EGF-mediated reduction of miR-92a-1-5p regulates HTR-8/SVneo cell invasion through the activation of MAPK8 and FAS, leading to a subsequent increase in MMP-2 and MMP-9 expression [14]. Researchers have also reported the significant downregulation of miR-92a-1-5p expression in the exosomes from prostate cancer patients [15, 16]. Nonetheless, researchers have yet to elucidate the specific effects of miR-92a-1-5p on osteosarcoma cell growth and metastasis, or the underlying mechanism. Further research will be required to confirm the role of miR-92a-1-5p in osteosarcoma and its impact on tumor progression and metastasis.

Tumor cells can induce the growth of sensory and sympathetic nerves within tumor tissue. This often leads to Cancer-Induced Bone Pain (CIBP) [17] in in situ bone tumors and tumor bone metastases, and becomes increasingly difficult to manage as tumor growth progresses [18]. The underlying mechanisms of CIBP encompass various neuropathological processes, including inflammation, ischemia, compression, or injury [19]. Recent studies have reported on the close relationship between CIBP and elevated nerve growth factor (NGF) expression levels [19]. NGF is an important neurotrophic factor promoting the growth of sensory and sympathetic nerves. It is highly expressed in the tumor microenvironment (TME) and interacts with various cells to facilitate cancer progression [20]. Tropomyosin receptor kinase A (TrkA) serves as a high-affinity catalytic receptor for NGF. In its function as a kinase, TrkA orchestrates various NGF effects, including neuronal differentiation, neural proliferation, nociceptor response, the inhibition of programmed cell death, cell metastasis, and tumor progression [20, 21]. Researchers have demonstrated that inhibiting NGF or its receptor (TrkA) significantly retards tumor development, reduces pain-related activity, and ameliorated bone damage in sarcoma. The NGF/TrkA axis has also been implicated in the metastasis of various tumors, including breast cancer [22], lung cancer [23], colon cancer [24], pancreatic cancer [25], and prostate cancer [20]. In recent years, medicinal chemists have made considerable advances in our understanding of TRK inhibitors. Larotrectinib (LOXO-101) is an highly selective small molecule inhibitor of the entire tropomyosin receptor kinase (TRK) family, including TrkA, TrkB, and TrkC. It has received FDA-approval for use in the treatment of TRK fusion-positive cancers, such as colon cancer [26], breast cancer [27], and lung cancer [28]. Despite the important role of the NGF/TrkA axis in tumor metastasis, researchers have yet to identify the specific molecular mechanisms by which it acts on osteosarcoma or the effectiveness of larotrectinib in this context. Further advances in understanding these mechanisms could pave the way for the development of novel targeted therapies for osteosarcoma.

## Materials and methods

### Analysis of mRNA expression profiles from the Gene Expression Omnibus (GEO) and Genomic Data Commons (GDC)

Gene expression data (GSE16088) were obtained from the GEO database, including comprehensive gene expression profiles from 6 normal tissue samples and 14 human osteosarcoma tumor tissue samples. Transcriptome profiles of osteosarcoma were accessed using the TCGA database via the GDC data portal (https://portal.gdc.cancer.gov/). RNA-Seq analysis was performed on 88 osteosarcoma samples to derive gene expression profiles of the neurotrophin family and tropomyosin receptor kinase (TRK) [29].

### Transwell cell migration and invasion assays

Cell migration was examined using 24-well Transwell culture plates (Costar, NY, USA) with pore size of 8-μm. For invasion assays, a 30 μL mixture of Matrigel and 0% DMEM (1:3) was pre-coated onto the filter membrane for 1 h. The osteosarcoma cells were either pre-transfected with siRNAs for 24 h or pretreated with designated inhibitors or the vehicle for 30 min. Approximately 1.5 × 10^4^ cells were seeded into the upper chamber with 200 μL of serum-free medium, while the lower chamber was filled with 300 μL of serum-free DMEM (for migration) and 10% DMEM (for invasion) containing NGF. After incubating in a humidified incubator under 5% CO_2_ at 37 °C for 18 h, the cells were fixed using 3.7% formaldehyde solution and stained using 0.05% crystal violet. Cell migration and invasion were assessed using a microscope by counting the number of cells that migrated to the lower chamber.

### Wound healing assays

Wound healing assays were performed using Ibidi cultures (Ibidi, Munich, Germany) inserted into 12-well plates and left to stand for 1 h. A total of 3 × 10^4^ osteosarcoma cells were inoculated in 100 mL of culture medium on each side of the culture insert. The culture insert was carefully removed after 24 h to create a cell-free area and scratch wound. After washing the sample with PBS, 1 mL of DMEM medium with 0.5% FBS was added. The progression of the scratch wound was observed and photographed using an inverted microscope at 0 and 24 h. The percentage of wound healing was quantified using ImageJ software using the following formula: [wound area (initial) - wound area (final)] / wound area (initial) × 100 [30].

### Colony formation assays

Cells were incubated in 6-well plates at a density of 2000 cells per well for 1 week, after which cell colonies were fixed using 4% paraformaldehyde and stained using 0.1% crystal violet solution (Sigma-Aldrich) for 15 min. Finally, the number of positive colonies containing over 50 cells was recorded.

### Establishing in vivo orthotopic xenograft model of osteosarcoma with lung metastasis

All animal experiments were conducted in strict adherence to protocols established by the Institutional Animal Care and Use Committee of Shin Kong Wu Ho-Su Memorial Hospital (Taipei, Taiwan) (IACUC approval number 112 SKH006). Male BALB/c nude mice were procured at 4 weeks of age from the Taipei National Laboratory Animal Center. The mice were randomly assigned to three groups: 143B, 143B/NGF, or 143B/NGF+Larotrectinib (*n* = 5 per group). Under 1.5–2.5% isoflurane anesthesia, 5 × 10^6^ 143B or 143B/NGF cells were implanted into the right tibia of each mouse using a solution comprising 50% serum-free DMEM and 50% Matrigel (total volume of 100 μL). The objective was to replicate in situ osteosarcoma growth. The 143B/NGF+Larotrectinib group was administered larotrectinib (50 mg/kg) orally three times a week for five weeks. Body weights and tumor volumes were assessed regularly. Euthanasia was performed via CO_2_ inhalation at six weeks. Tibia tumor and lung samples were collected, fixed in 10% formalin, and prepared for subsequent analysis [31].

### Immunohistochemical (IHC) staining

Human normal bone tissue arrays (BO244g; containing 11 cases of bone tissue and one osteosarcoma case) and human osteosarcoma tissue arrays (OS804d; containing 40 cases of osteosarcoma, and OS802c; containing 50 cases of osteosarcoma) were obtained from US Biomax, Inc (Rockville, MD, USA). The tissue specimens underwent rehydration before immunohistochemical staining using primary antibodies against NGF or MMP-2, which were diluted at a 1:250 ratio. After applying Biotin-labeled secondary antibodies, the antibody binding signal was visualized using the NovoLink Polymer Detection System (Leica Microsystems, Heidelberg, Germany) using 3,3’-diaminobenzidine with hematoxylin for counterstaining. Staining intensity was assessed from three images per slide by three independent pathologists using a scale ranging from 0 to 5 (0: negative, 1: low, 2: weak, 3: moderate, 4: strong, and 5: very strong). IHC scores were calculated by summing the intensity scores. The same procedure was performed on mouse lung tissues for comparative analysis.

### Statistical analysis

The data are presented as mean ± standard deviation (SD). Statistical analysis was performed using the two-tailed Student’s *t*-test to determine the significance of differences between groups, with a significance level set at 0.05.

Details pertaining to materials and cell cultures as well as the procedures and protocols used in cell counting kit-8 (CCK-8) assays, Western blot analysis, quantitative real-time polymerase chain reaction (qRT-PCR) assay, Small interfering RNA (siRNA) transfection, and MicroRNA (miRNA) database searches can be found in the Supplementary Information.

## Results

### Clinicopathological features and functions of NGF in human osteosarcoma

**Fig. 1:**
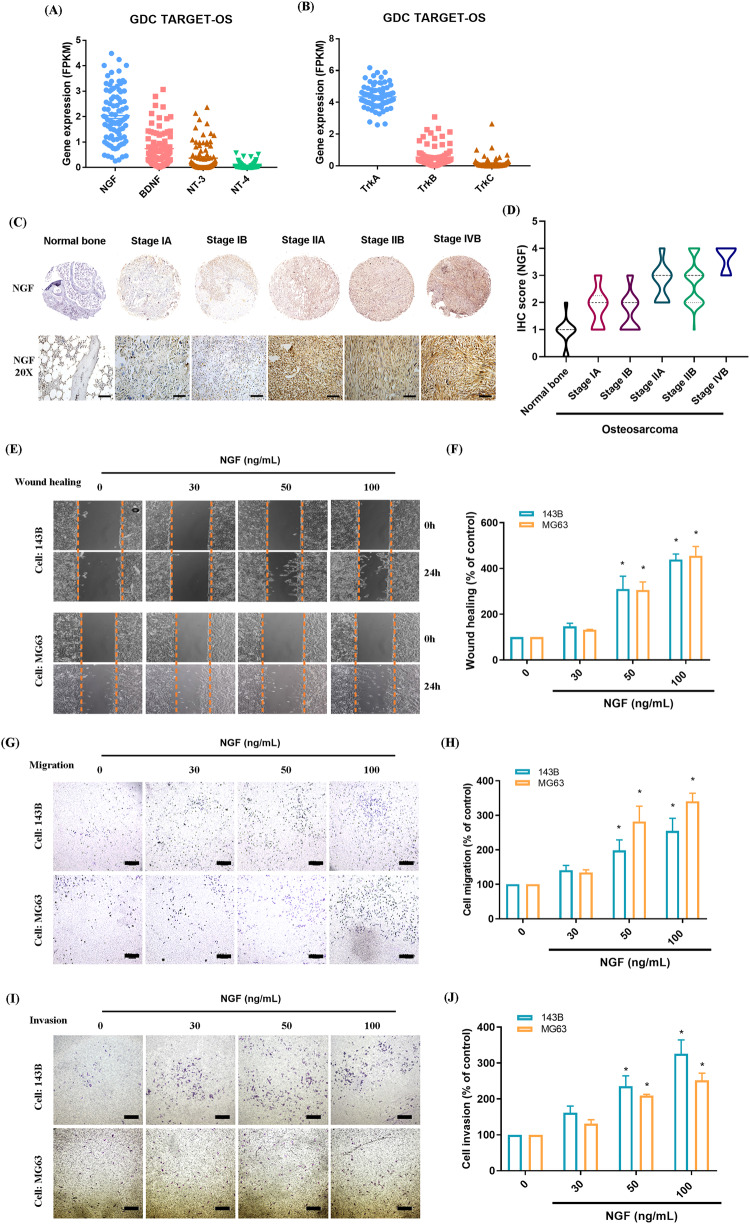
NGF is highly expressed in advanced osteosarcoma tissues and promotes cell migration. **A**, **B** NGF and TrkA family members mRNA expression in osteosarcoma tissue was analyzed in samples from the GDC data portal. **C**, **D** Normal bone and different stages of osteosarcoma specimens were stained with NGF antibody (scale bar 100 μm) for IHC, followed by quantification. **E**–**J** Osteosarcoma cells were incubated with different concentrations of NGF (30–100 ng/mL) for 18 or 24 h and cell migration and invasion were examined by the wound healing and the Transwell assay (scale bar 500 μm). All experiments were repeated 3 to 5 times. *p < 0.05 compared with the control group.

The NGF/TrkA axis is closely associated with the progression of many cancers and poor clinical outcomes [24, 32]. Analysis was performed on 88 osteosarcoma tissue samples from the Genomic Data Commons (GDC) data portal with the aim of establishing NGF expression levels and elucidating its role in osteosarcoma. In osteosarcoma samples, NGF and TrkA mRNA expression levels were higher than those of BDNF, NT-3, NT-4, TrkB, or TrkC (Fig. 1A, B). The results obtained from commercially available tissue arrays revealed a strong correlation between NGF expression levels and the clinical stage of osteosarcoma (Fig. 1C, D). In our exploration of the impact of NGF on cell motility in osteosarcoma cell lines (143B and MG63), it was observed that NGF treatment led to dose-dependent improvements in wound healing, migration, and invasion abilities (Fig. 1E–J). In summary, the upregulation of NGF in osteosarcoma is positively correlated with the clinical stage of the disease and enhances the metastatic potential.

### NGF is positively correlated with MMP-2 in human osteosarcoma tissue and enhances MMP-2-dependent migration of osteosarcoma cells

**Fig. 2:**
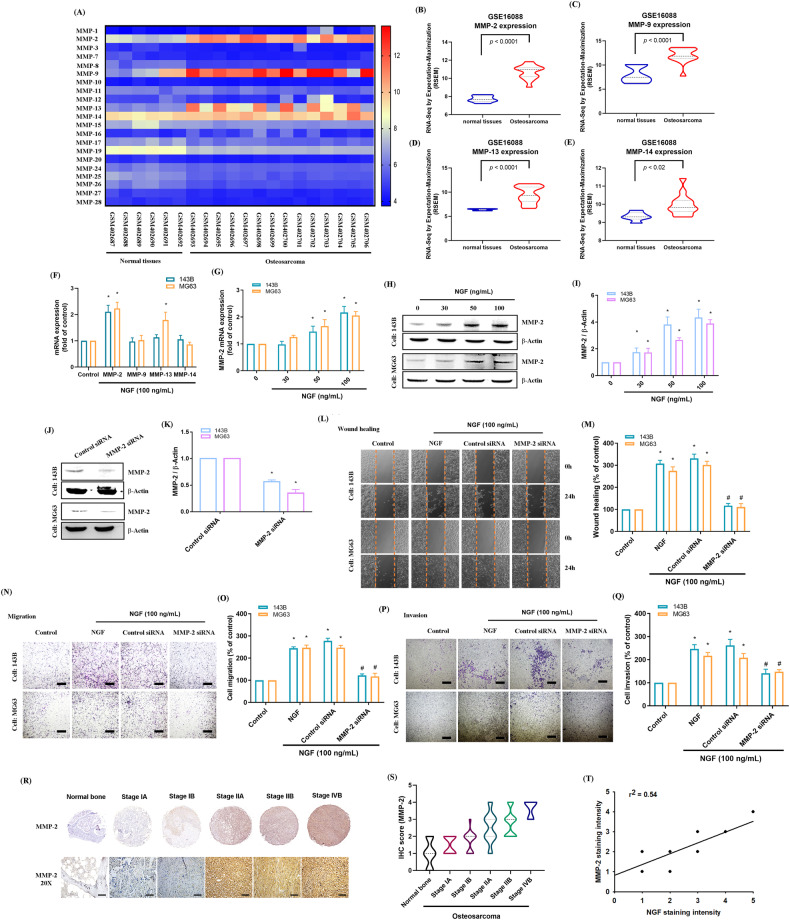
MMP-2 is highly expressed in osteosarcoma tissues, and NGF promotes MMP-2-dependent cell migration in human osteosarcoma. **A**–**E** MMPs mRNA expression in normal and osteosarcoma tissue was analyzed in samples from the GEO GSE16088 dataset. **F** Osteosarcoma cells were incubated with NGF for 24 h, before determining 4 potential MMPs candidates by qPCR. **G**–**I** Osteosarcoma cells were incubated with varying concentrations of NGF (30, 50, or 100 ng/mL) for 24 h, and the levels of MMP-2 expression were examined by qPCR and Western blot assays. **J**–**Q** Osteosarcoma cells were transfected with MMP-2 siRNAs for 24 h, then stimulated with NGF, and cell migration, invasion, as well as levels of MMP-2 expression, were examined by the wound healing, Transwell, and Western blot assays (scale bar 500 μm). **R**, **S** Normal bone and osteosarcoma specimens were subjected to IHC staining with MMP-2 antibody (scale bar 100 μm), then quantified. **T** A positive correlation between NGF and MMP-2 protein expression in human osteosarcoma tissue. All experiments were repeated 3 to 5 times. *p < 0.05 compared with the control group; ^#^*p* < 0.05 com*p*ared with the NGF-treated group.

Numerous studies have confirmed that MMP expression levels are strongly correlated with the poor prognosis after metastasis in osteocarcinoma. They have also established the mechanisms by which MMP promotes osteosarcoma invasion, lung metastasis, and antitumor activity [6, 33]. In the current study, we used the GEO dataset (GSE16088) to analyze the expression of MMPs in normal bone tissues and osteosarcoma tissues. The results revealed that the expression levels of MMP-2, -9, -13, and -14 mRNA are significantly higher in osteosarcoma tissue than in normal bone tissues (Fig. 2A–E). We also examined the impact of NGF on the expression of four MMPs in osteosarcoma cells. NGF induced MMP-2 expression in both osteosarcoma cell lines as well as increases in MMP-2 mRNA and protein levels in a dose-dependent manner (Fig. 2F–I). The functional implications were examined by performing MMP-2 siRNA transfection to reduce the effects of NGF on wound healing and migratory and invasion abilities (Fig. 2J–Q). This analysis revealed that NGF promotes osteosarcoma cell metastasis by upregulating MMP-2 expression. Immunohistochemical (IHC) staining of the osteosarcoma tissue array using MMP-2 antibodies revealed a notable correlation between MMP-2 expression and the clinical stage of the disease (Fig. 2R, S). We also observed a positive correlation between NGF and MMP-2 expression in osteosarcoma tissue (Fig. 2T). These observations suggest that both NGF and MMP-2 are positively correlated with the clinical stage of osteosarcoma and that NGF enhances MMP-2-dependent metastasis of osteosarcoma cells.

### MEK/ERK signaling moderates the effects of NGF in promoting MMP-2-mediated wound healing and osteosarcoma cell migration

**Fig. 3:**
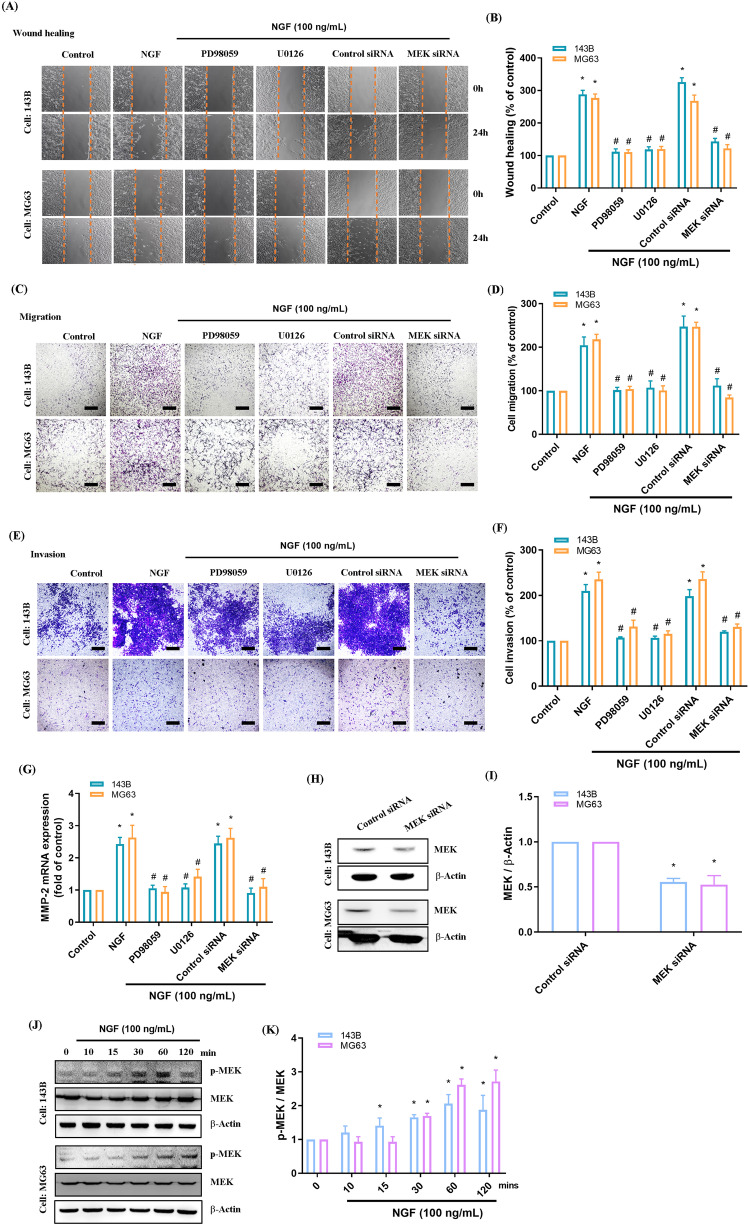
The MEK pathway mediates NGF-induced MMP-2 expression and cell motility. **A**–**F** Osteosarcoma cells were incubated with MEK inhibitors (PD9059 and U0126) for 30 min or transfected with a MEK siRNA for 24 h, then stimulated with NGF, and cell migration and invasion were examined by the wound healing and the Transwell assay (scale bar 500 μm). **G** Cells were treated with MEK inhibitors for 30 min or transfected with a MEK siRNA for 24 h, then stimulated with NGF for 24 h. The levels of MMP-2 mRNA expression were examined by qPCR assays. **H**, **I** Cells were transfected with a MEK siRNA for 24 h, and levels of MEK protein expression were examined by Western blot. **J**, **K** Osteosarcoma cells were incubated with NGF for the indicated time intervals; MEK phosphorylation was examined by Western blot. All experiments were repeated 3 to 5 times. *p < 0.05 compared with the control group; ^#^*p* < 0.05 com*p*ared with the NGF-treated group.

**Fig. 4:**
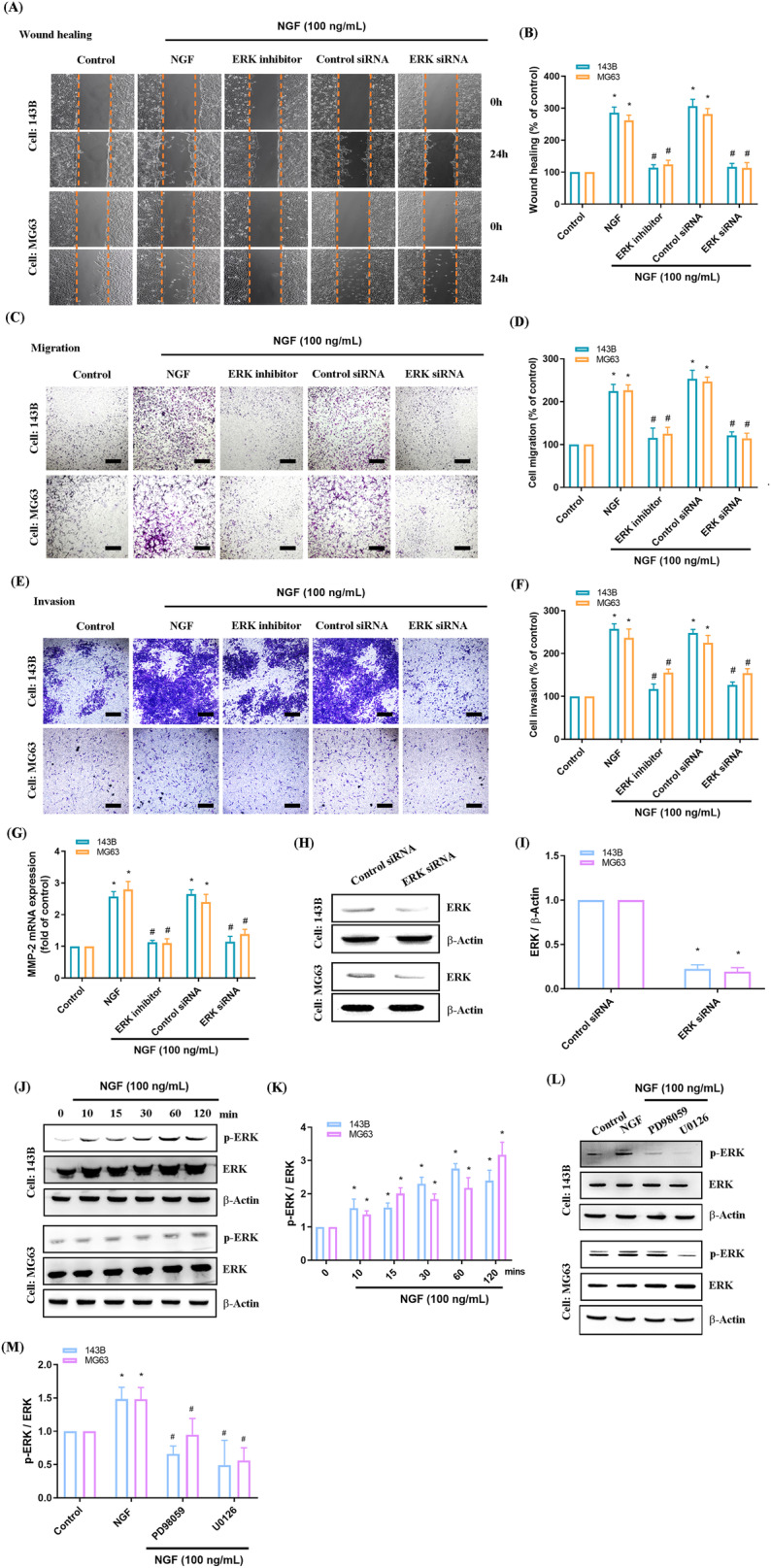
The ERK pathway is involved in NGF-induced osteosarcoma MMP-2 expression and cell migration. **A**–**F** Osteosarcoma cells were treated with an ERK inhibitor for 30 min or transfected with an ERK siRNA for 24 h. Subsequently, the cells were stimulated with NGF, and cell migration and invasion were examined using the wound healing and the Transwell assay (scale bar 500 μm). **G** Cells were incubated with ERK inhibitors for 30 min or transfected with an ERK siRNA for 24 h, followed by stimulation with NGF for 24 h. The levels of MMP-2 mRNA expression were examined using qPCR assays. **H**, **I** Cells were transfected with an ERK siRNA for 24 h, and the levels of ERK protein expression were examined using Western blot. **J**, **K** Osteosarcoma cells were incubated with NGF for the indicated time, and the phosphorylation of ERK was examined using Western blot. **L**, **M** Osteosarcoma cells were pretreated with MEK inhibitors for 30 min and then stimulated with NGF for 15 min; the phosphorylation of ERK was examined using Western blot. All experiments were repeated 3 to 5 times. *p < 0.05 compared with the control group; ^#^*p* < 0.05 com*p*ared with the NGF-treated group.

The MEK/ERK signaling pathway is associated with metastasis in various cancer cell lines, including osteosarcoma [34, 35]. In the current study, we explored the involvement of MEK/ERK signaling in moderating the NGF-induced promotion of MMP-2-mediated wound healing, as well as the migratory and invasion abilities of osteosarcoma cells. This was achieved by using specific inhibitors targeting MEK (PD98059 and U0126) and ERK (ERK inhibitor) as well as MEK and ERK siRNAs. MEK and ERK inhibition significantly attenuated the effects of NGF in promoting MMP-2 mRNA expression and migratory and invasion activity in osteosarcoma cells (Figs. 3A–I, 4A–I).

We determined that NGF stimulation led to MEK and ERK phosphorylation in osteosarcoma cells (Figs. 3J, K, 4J, K). We also determined that MEK inhibitors reversed NGF-mediated ERK phosphorylation (Fig. 4L, M). These findings suggest that MEK/ERK signaling is involved in mediating the effects of NGF on MMP-2-mediated wound healing and cell migration in osteosarcoma cells.

### The miR-92a-1-5p/MMP-2 axis regulates NGF-enhanced wound healing and the migration of osteosarcoma cell

**Fig. 5:**
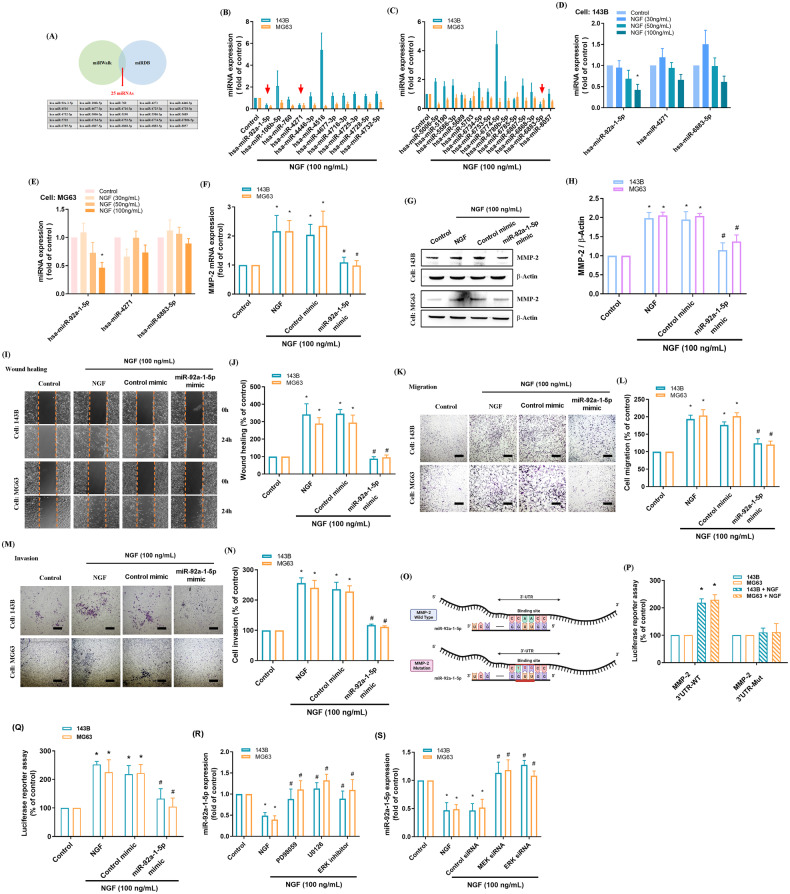
NGF down-regulates the expression of miR-92a-1-5p through the MEK/ERK signaling pathway, increases the expression of MMP-2 and promotes the migration of osteosarcoma cells. **A** An analysis of 2 miRNA prediction databases predicted 25 miRNAs that bind with MMP-2 3’-UTR. **B**, **C** The osteosarcoma cells were treated with NGF and miRNAs were screened using a qPCR assay. **D**, **E** Osteosarcoma cells were treated with different concentration of NGF (30, 50, 100 ng/mL) for 24 h and miRNAs expression were examined by qPCR. **F**–**N** Osteosarcoma cells were transfected with miR-92a-1-5p mimic or control mimic for 24 h, then stimulated with NGF for 24 h. The levels of MMP-2 and cell migration were examined by qPCR, Western blot, wound healing, and the Transwell migration (scale bar 500 μm). **O** The MMP-2 3’-UTR wild-type and mutant contained the miR-92a-1-5p binding site. **P** Osteosarcoma cells were transfected with the MMP-2 3’-UTR wild-type or mutant plasmid for 24 h, then stimulated with NGF for 24 h, and relative luciferase activity was measured. **Q** Osteosarcoma cells were co-transfected with miR-92a-1-5p mimic and MMP-2 3’-UTR wild-type for 24 h, then stimulated with NGF for 24 h, and relative luciferase activity was measured. **R**, **S** Osteosarcoma cells were pretreated with MEK and ERK inhibitors for 30 min, or transfected siRNA for 24 h, then stimulated with NGF for 24 h, and the miR-92a-1-5p expression was quantified by qPCR. All experiments were repeated 3 to 5 times. *p < 0.05 compared with the control group; ^#^*p* < 0.05 com*p*ared with the NGF-treated group.

Numerous studies have confirmed that miRNAs play key roles in the progression, metastasis, and proliferation of cancer cells. They can also be used as prognostic markers [36, 37]. We initially used two online databases, miRWalk and miRDB, to predict miRNA targets within the 3’-UTR region of MMP-2 mRNA. This analysis revealed 25 candidate miRNAs (Fig. 5A). Treating osteosarcoma cells (143B and MG63) with NGF (100 ng/mL) revealed the significant downregulation of three miRNAs, namely miR-92a-1-5p, miR-4271, and miR-6883-5p (Fig. 5B, C). In both osteosarcoma cell lines, exposure to NGF at various concentrations (30, 50, or 100 ng/mL) revealed the concentration-dependent suppression of miR-92a-1-5p expression (Fig. 5D, E). Transfection of osteosarcoma cells with a miR-92a-1-5p mimic significantly reduced NGF-induced MMP-2 mRNA and protein expression, as well as the cell migration and invasion abilities (Fig. 5F–N). We also investigated the impact of miR-92a-1-5p binding to the MMP-2 3’-UTR on MMP-2 transcription by constructing MMP-2 WT 3’-UTR and MMP-2 MUT 3’-UTR luciferase reporter plasmids (Fig. 5O). NGF was shown to enhance the luciferase activity of the wild-type MMP-2 3’-UTR, whereas the mutant MMP-2 3’-UTR generated no such response (Fig. 5P). Additionally, pre-transfection with a miR-92a-1-5p mimic significantly suppressed the enhancement of MMP-2 luciferase activity induced by NGF (Fig. 5Q). We subsequently investigated the role of MEK and ERK signaling in regulating the NGF-induced repression of miR-92a-1-5p synthesis. Pre-treating osteosarcoma cells with MEK and ERK inhibitors or their respective siRNAs reversed the effects of NGF in repressing miR-92a-1-5p expression (Fig. 5R, S). These findings confirm that in human osteosarcoma cells, miR-92a-1-5p controls MMP-2 expression and cell migration by binding to the 3’-UTR of the MMP-2 gene via the MEK/ERK pathway.

### Larotrectinib inhibits NGF-induced metastasis of osteosarcoma cells

**Fig. 6:**
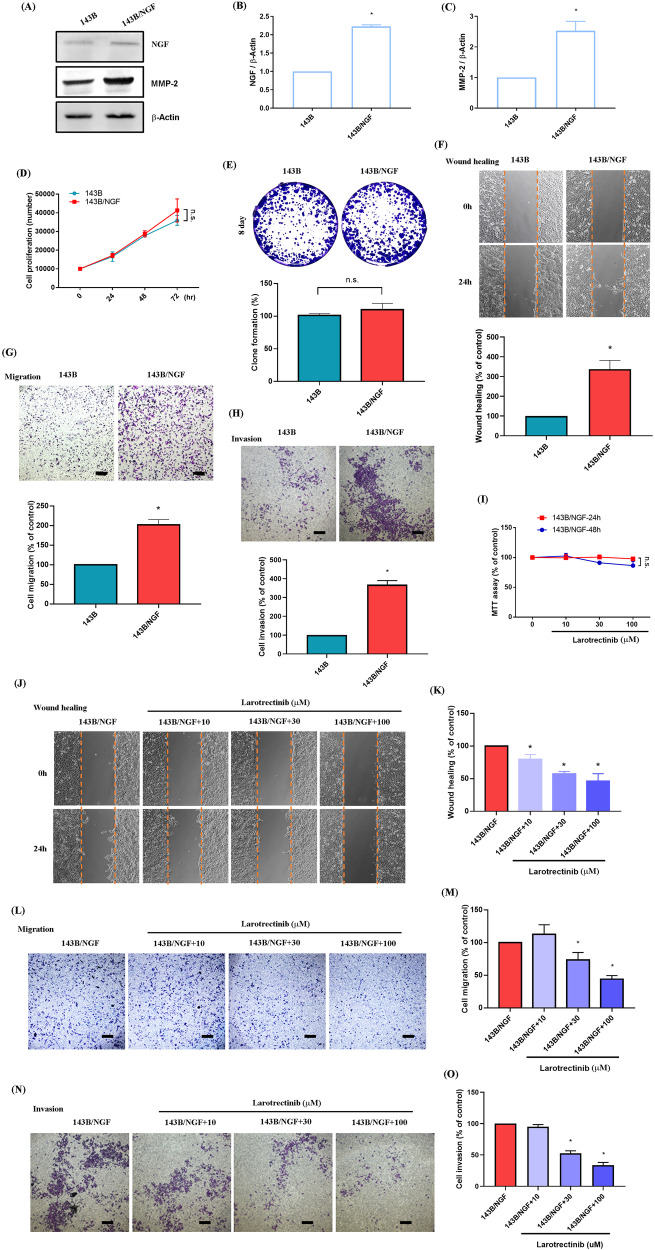
Larotrectinib suppresses NGF-induced osteosarcoma cells migration in vitro*.* **A**–**C** 143B cells were transfected with an empty vector or an NGF-overexpressing vector (143B/NGF). The overexpression efficiency was detected by immunoblotting. **D**–**H** The proliferation, migration, and invasion of 143B and 143B/NGF osteosarcoma cells were analyzed using colony formation, CCK-8 assay, wound healing, and the Transwell assays (scale bar 500 μm). **I** 143B/NGF cells were incubated with different concentrations of larotrectinib (10, 30, 100 μM) for 24 and 48 h, and cell viability was measure cell viability using MTT. **J**–**O** Osteosarcoma cells were treated with larotrectinib for 30 min. Following this, the cells were stimulated with NGF, and cell migration and invasion were assessed using the wound healing assay and the Transwell assay (scale bar 500 μm). All experiments were repeated 3 to 5 times. *p < 0.05 compared with the 143B group.

The mechanism of endogenous NGF in osteosarcoma was elucidated by establishing an overexpressing NGF osteosarcoma cell line (143B/NGF) to confirm protein expression using Western blot analysis (Fig. 6A–C). The proliferative effects were examined using colony formation assays and cell proliferation experiments. These results revealed no significant difference between 143B and 143B/NGF cells in trms of cell proliferation (Fig. 6D, E). However, in wound healing, transwell migration, and invasion assays, the migration ability of 143B/NGF cells was significantly higher than that of 143B cells, thereby demonstrating that endogenous NGF can control the motility of osteosarcoma cells (Fig. 6F–H). Larotrectinib is an orally administered ATP-competitive inhibitor of the tropomyosin receptor kinase (TRK) family (TRKA, B, and C), which blocks the expression of NGF [26]. We first examined the cytotoxic effects of larotrectinib on 143B/NGF cells. In MTT assays, larotrectinib concentrations of 10, 30, and 100 μM had no effect on cell viability over periods of 24 or 48 h (Fig. 6I). In wound healing, transwell migration, and invasion experiments, larotrectinib significantly inhibited the migration and invasion ability of 143B/NGF cells in a dose-dependent manner (Fig. 6J–O). These results confirm that endogenous NGF can significantly enhance the migration ability of osteosarcoma cells, the effects of which can be suppressed by larotrectinib.

### Larotrectinib reverses lung metastasis of osteosarcoma cells in vivo by blocking NGF overexpression

**Fig. 7:**
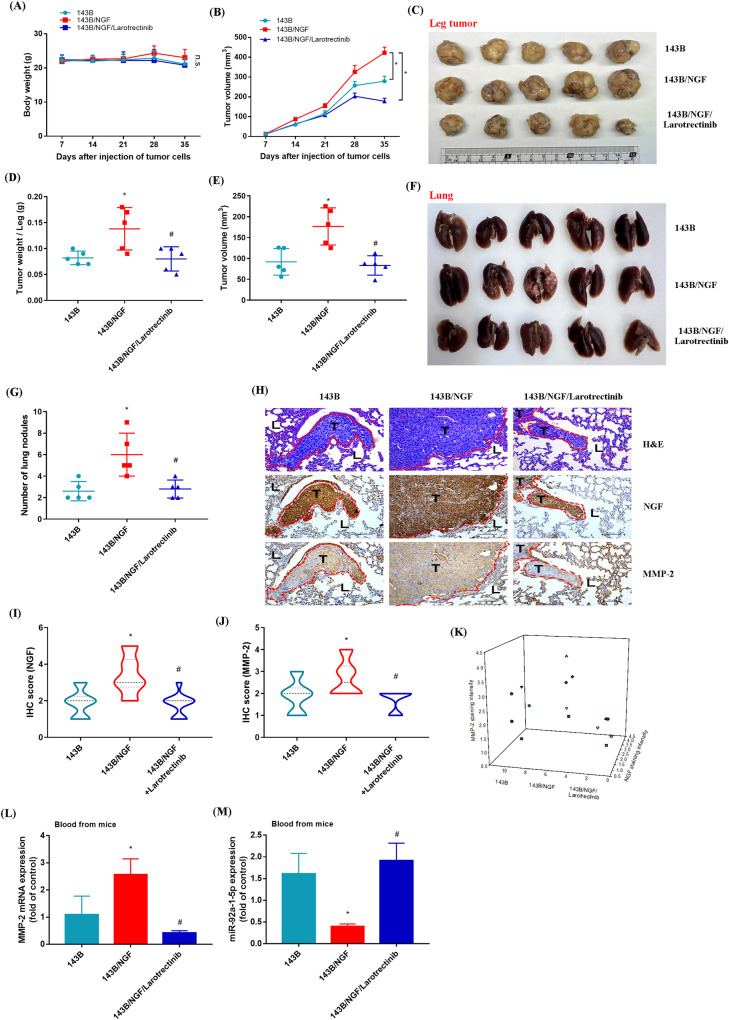
Larotrectinib inhibits NGF-induced osteosarcoma metastasis to the lungs in vivo. **A**, **B** Mice were injected with three groups of cells (143B, 143B/NGF, or 143B/NGF+Larotrectinib) (*n* = 5 per group). Mouse body weight and tumor size were measured at indicated time intervals. **C**–**G** After six weeks, the mice were humanely sacrificed, and the leg tumor and lung tissue were excised, photographed, and quantified. **H**–**K** IHC analysis was performed on the protein expression levels of NGF and MMP-2 in lung tumors, and a positive correlation was observed between the protein expressions of NGF and MMP-2. L, lung tissue; T, tumor. (**L**, **M**) Mouse blood samples were analyzed for levels of human MMP-2 mRNA and miR-92-1-5p expression by qPCR. *p < 0.05 compared with the 143B group; ^#^*p* < 0.05 com*p*ared with the 143B/NGF group.

In a previous study, we developed an orthotopic mouse model to explore the mechanisms underlying the lung metastasis of bone cancer [38]. In this study, osteosarcoma cells (143B and 143B/NGF) were orthotopically implanted into the right tibia of mice, after which the body weight and tumor volume was recorded weekly. The tumor volume of the overexpressing NGF cell line (143B/NGF) was significantly larger than that of the original 143B cell line. The oral administration of larotrectinib (50 mg/kg) three times a week significantly inhibited 143B/NGF tumor growth without affecting the body weight of the mice (Fig. 7A, B). Mice were sacrificed 6 weeks after tumor implantation to enable further analysis of tumor volume in the right tibia and the occurrence of lung metastasis. We found that 143B/NGF cells were more likely than 143B cells to grow and undergo lung metastasis. We also determined larotrectinib treatment inhibited both effects (Fig. 7C−G). IHC results of mouse lungs in the 143B/NGF orthotopic model revealed a significantly positive correlation between NGF and MMP-2 protein expression levels (Fig. 7H−K). These results confirm that NGF promotes osteosarcoma metastasis to the lungs of mice. We also analyzed the expression of cancer cells in mouse blood. qPCR results revealed that MMP-2 expression was higher in the 143B/NGF group than in the 143B group. Larotrectinib was shown to inhibit the expression of the MMP-2 gene in the 143B/NGF group, while promoting the expression of miR-92a-1-5p (Fig. 7L, M). These results suggest that NGF promotes osteosarcoma metastasis by promoting MMP-2 expression via the inhibition of miR-92a-1-5p expression. These results also demonstrate that larotrectinib suppresses these effects.

## Discussion

Osteosarcoma patients often experience Cancer-induced Bone Pain (CIBP), due to the abundant distribution of sensory nerves in the bones [39]. Studies have confirmed that the secretion of NGF by tumors induces nerve regeneration, while promoting tumor cell survival, cell motility, and invasion [25]. NGF is an important neurotrophic factor shown to promote tumor cell survival, cell motility, and invasion [25]. NGF stimulation leads to the over-activation of the TRKA receptor kinase domain and the activation of downstream signal pathways, including RAS-MAPK, PI3K-AKT, JAK2-STAT3, PLC γ, and Hippo pathway. Note that these pathways are involved in diverse cellular processes, including tumor cell proliferation, invasion, epithelial-mesenchymal transition (EMT), perineural invasion (PNI), drug resistance, and cancer pain. Exosomes have been shown to promote the secretion of cytokines (including NGF), thereby fostering the formation of inflammatory and immunosuppressive microenvironments. Exosomes from H1299 have also been shown to affect the corresponding metabolic pathways, thereby facilitating the establishment of a pre-metastatic niche for brain metastases in cases of lung cancer. NGF derived from has been shown to activate the TrkA/ERK/ELK1/ZEB1 signaling pathway in colon cancer cells. This activation boosts the expression of exosomal miR-21-5p, leading to increased proliferation and metastasis. These findings collectively underscore the importance of NGF in tumor metastasis. This study found that NGF induces metastasis in osteosarcoma. We also determined that NGF expression is positively correlated with disease progression. This insight means that NGF could potentially be a therapeutic target in clinical research on osteosarcoma.

Cancer cell lung metastasis is one of the main reasons for the poor prognosis among osteosarcoma patients [33]. Extensive research has implicated MMPs in cancer progression [2]. MMP-2, MMP-7, MMP-9, and MMP-14 have been linked to the progression and metastasis of osteosarcoma [40, 41]. Xu et al. reported that berberine inhibits the migration and invasion of osteosarcoma cells by downregulating the expression of MMP-2 and MMP-9s [42]. Aripiprazole has been shown to inhibit metastatic behavior in human osteosarcoma cells by reducing MMP-2 and MMP-9 expression levels and activity [40]. In cases of esophageal squamous cell carcinoma (ESCC), silencing circ_0046534 has been shown to inhibit the growth and metastasis of ESCC via the miR-339-5p/MMP2 pathway [43]. These studies collectively highlight the crucial role of MMP-2 in tumor metastasis, particularly in cases of osteosarcoma. In the current study, we confirmed that NGF promotes the migration of osteosarcoma cells by mediating the production of MMP-2, rather than other MMPs. We also observed high expression levels of NGF and MMP-2 proteins in late-stage tumor samples in an osteosarcoma tissue array. Note that these expression levels were positively correlated with osteosarcoma progression. These findings suggest a close association between NGF and MMP-2. The current study was based on the hypothesis that MMP-2 is an important factor in the NGF-induced migration of osteosarcoma. Our research identified the targeting of MMP-2 depletion as a novel therapeutic approach to the treatment of osteosarcoma metastasis.

The fact that MEK/ERK signaling pathways are widely involved in cancer progression and metastasis has made them main targets in research on the treatment of osteosarcoma [44, 45]. Lipocalin-2 suppresses osteosarcoma cell metastasis by inhibiting MET expression via the MEK/ERK pathway [46]. By targeting the MEK/ERK signaling pathway, Cobimetinib has been shown to inhibit metastasis in osteosarcoma and enhance the efficacy of doxorubicin in osteosarcoma models. These results confirm the therapeutic value of the MEK/ERK pathway in the clinical management of osteosarcoma [44]. In the current study, NGF promoted the phosphorylation of MEK and ERK, while pharmacological inhibitors and siRNA targeting MEK and ERK inhibited NGF-induced MMP-2 expression and the migration of osteosarcoma cells. The fact that MEK inhibitors also suppress NGF-induced ERK phosphorylation suggests that MEK-dependent ERK activation mediates the NGF-promoted synthesis of MMP-2 and osteosarcoma cell motility. These results suggest that the MEK/ERK signaling pathway plays a critical regulatory role in NGF-induced osteosarcoma metastasis.

MiRNAs are single-stranded RNAs (20-24 nucleotides in length) that lack coding capacity [16, 47]. Their function is to inhibit the translation or degradation of target mRNA, thereby regulating gene expression post-transcriptionally [29]. Previous studies have confirmed that 13 miRNAs in serum are involved in the regulation of osteosarcoma progression, and can be used as diagnostic and prognostic markers [47]. This suggests that miRNA could also be a target for the treatment of osteosarcoma. Despite reports that miR-92a-1-5p disrupts bone homeostasis and promotes bone metastasis in prostate cancer [16], its role in osteosarcoma remains unclear. In the current study, bioinformatics analysis identified MMP-2 as a direct target of miR-92a-1-5p. This study is the first to demonstrate a potential miR-92a-1-5p/MMP-2 regulatory axis involved in metastasis of osteosarcoma. This research also provided evidence that NGF significantly downregulates the expression of miR-92a-1-5p, thereby promoting MMP-2 expression and osteosarcoma metastasis. Treating osteosarcoma cells with MEK and ERK inhibitors or siRNA was shown to reverse the inhibitory effect of NGF on miR-92a-1-5p expression. These findings suggest that NGF enhances MMP-2 expression by suppressing miR-92a-1-5p and promoting osteosarcoma metastasis via the MEK/ERK signaling cascade. Nevertheless, further investigations are warranted to determine whether these signaling cascades modulate the expression of miR-92a-1-5p via transcriptional or post-transcriptional mechanisms.

First-generation tropomyosin receptor kinase (TRK) inhibitors, larotrectinib and entrectinib, are ATP-competitive inhibitors that bind specifically effective in the treatment of various cancers, including infantile fibrosarcoma [48], anaplastic thyroid cancer [49]. Robert et al. demonstrated that inhibiting compensatory neuronal innervation with LOXO-101 (a Trk-NGF inhibitor) further reduced PDAC tumor growth. Our findings provide further evidence that larotrectinib impedes NGF-induced osteosarcoma cell migration in vitro and lung metastasis in vivo. These findings underscore the potential of larotrectinib in attenuating the osteosarcoma cell migration induced by NGF. Further research may unveil novel approaches to the treatment of osteosarcoma.

**Fig. 8:**
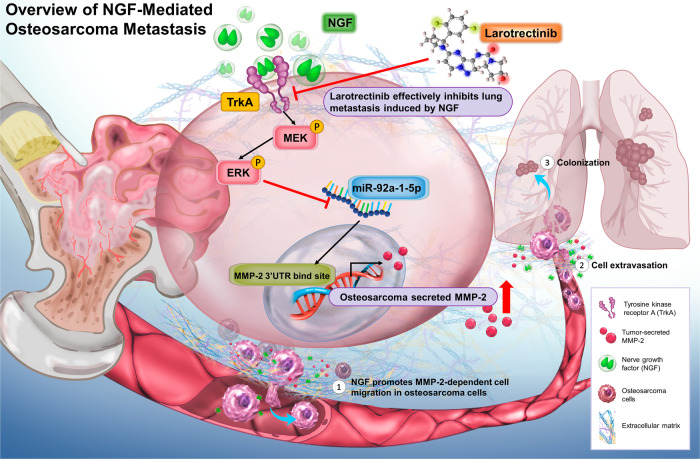
The schematic diagram illustrates the mechanism of NGF in osteosarcoma metastasis. NGF promotes MMP-2-dependent osteosarcoma cell migration by inhibiting the effects of miR-92a-1-5p through the MEK/ERK signaling cascade, while larotrectinib effectively suppresses NGF-induced cell migration.

In summary, this study confirmed that NGF suppresses the synthesis of miR-92a-1-5p via the MEK/ERK signaling cascade, thereby promoting the MMP-2-dependent migration of osteosarcoma cells. Larotrectinib exhibited excellent tumor-targeting effects by inhibiting the NGF-induced growth of tumors and lung metastasis (Fig. 8). Targeting NGF expression is a promising approach to the development of novel treatments for metastatic osteosarcoma.

### Supplementary information

Supplementary Materials and Methods

Full western blots

## Data Availability

This published article and its supplementary information files include all data generated or analyzed during this study. The data generated in this study are available upon reasonable request from the corresponding author.
