# Supplementary material for: Targeting nerve growth factor-mediated osteosarcoma metastasis: mechanistic insights and therapeutic opportunities using larotrectinib
Source: Cell Death Dis. 2024 May 30;15(5):381. doi: 10.1038/s41419-024-06752-0 (PMC11139949; doi:10.1038/s41419-024-06752-0)
Supplement: Supplementary file 1 — Supplementary Materials and Methods [file 41419_2024_6752_MOESM1_ESM.doc]

**Supplementary Materials and Methods**

Materials

The following antibodies and reagents were utilized in this study: Rabbit polyclonal antibodies specific for p-MEK1/2 (Ser221) (166F8) (Catalog No: 2338) were purchased from Cell Signaling Technology (Danvers, MA, USA). MEK-1 (H-8) (Catalog No: SC-6250), p-ERK (E-4) (Catalog No: SC-7383), ERK2 (D-2) (Catalog No: SC-1647), and NGF (E-12) (Catalog No: SC-365944) antibodies, as well as all siRNAs, were obtained from Santa Cruz Biotechnology (Santa Cruz, CA, USA). MMP-2 (Catalog No: MAB3308) and β-actin (Catalog No: A5441) antibodies were purchased from Sigma-Aldrich (St. Louis, MO, USA). The miR-92a-1-5p mimic, miRNA control, Lipofectamine 2000, and Trizol were obtained from Life Technologies (Carlsbad, CA, USA). Dulbecco's Modified Eagle Medium (DMEM), fetal bovine serum (FBS), and all other cell culture reagents were purchased from Gibco-BRL Life Technologies (Grand Island, NY, USA). All other chemicals were obtained from Sigma-Aldrich (St. Louis, MO, USA).

Cell culture

Human osteosarcoma cell lines (MG-63 and 143B) were procured from the American Type Cell Culture Collection (ATCC) (Manassas, VA, USA). We transfected 143B cells with NGF overexpression plasmids using Lipofectamine 2000. After 48 hours, the medium was replaced, and then 200 µg/mL G418 (Cyrusbioscience, Inc., Taiwan) was added to select stable transfectants. The selection medium was refreshed every 3 days. After 2 weeks of G418 selection, clones of resistant cells were isolated (143B/NGF). All osteosarcoma cells were cultured in DMEM medium supplemented with 10% FBS and antibiotics. The cells were maintained in a humidified incubator at 37°C with 5% CO_2_.^1^.

Cell Counting Kit-8 (CCK-8) assay

To assess cell viability, we used the CCK-8 kit. Osteosarcoma cells were seeded in 96-well plates at a density of 5×10^3^ cells per well, and treated with different concentrations of larotrectinib (10, 30, and 100 μM) for 24 hours or 48 hours. Then 10 μL of CCK-8 reagent was added per well and further incubated at 37°C for 1 hour. Finally, cell viability was determined by measuring the absorbance at 450 nm^2^.

Western blot analysis

To prepare cellular lysates, we followed established previously described protocols^2, 3^. The resulting proteins were separated by sodium dodecyl sulfate-polyacrylamide gel electrophoresis (SDS-PAGE) and subsequently transferred onto polyvinylidene fluoride (PVDF) membrane filters. The PVDF membranes were blocked with Tris-Buffered Saline-Tween 20 (TBST) containing 4% non-fat milk at room temperature for 1 hour. Afterward, the membranes were probed with primary antibodies against p-MEK, MEK, p-ERK, ERK, MMP-2, NGF, or β-actin for 1 hour at room temperature, followed by three 5-minute washes with TBST. The membranes were then incubated with HRP-conjugated anti-rabbit or anti-mouse secondary antibodies for 1 hour at room temperature. All western blots within a panel are from the same experiment, and all blots were processed in parallel. Finally, the blot membranes were visualized using a Fujifilm LAS-3000 imaging system (Fujifilm, Tokyo, Japan).

Quantitative real-time polymerase chain reaction (qRT-PCR)

Total RNA was extracted from osteosarcoma cells (MG63 and 143B) using the TRIzol kit (MDBio Inc., Taipei, Taiwan). Subsequently, cDNA synthesis was carried out using the Invitrogen reverse transcription kit (Carlsbad, CA, USA). 2 μL of cDNA template, sequence-specific primers, and SYBR Green PCR Master Mix (Thermo Scientific, Waltham, MA) were included in a total volume of 20 μL when analyzed using real-time PCR. Relative gene expression was calculated using a 2-ΔΔCt method by normalization with glyceraldehyde 3-phosphate dehydrogenase (GAPDH). The qPCR analysis of miR-92a-1-5p expression was performed on the StepOnePlus sequence detection system using the TaqMan MicroRNA Reverse Transcription Kit, and the results were normalized to U6 expression^3^. The sequences of the primers were as follows: MMP-2 forward 5’-GATACCCCTTTGACGGTAAGGA-3′, and MMP-2 reverse 5’-CCTTCTCCCAAGGTCCATAGC-3’. GAPDH forward 5′-ACCACAGTCCATGCCATCAC-3′, and GAPDH reverse 5′-TCCACCACCCTGTTGCTGTA-3′. hsa-miR-92a-1-5p 5’-AGGTTGGGATCGGTTGCAATGCT-3’.

Small interfering RNA (siRNA) transfection

All silencing siRNA, including control siRNA, were purchased from Santa Cruz Biotechnology (Santa Cruz, USA). Transient transfection of siRNA was performed using Lipofectamine 2000 transfection reagent (Invitrogen, Carlsbad, CA) following the manufacturer’s protocol. Osteosarcoma cells (5 × 10^5^ cells per well) were seeded in a 6-well plate and cultured for 16 hours until reaching 80% confluence. One hour before transfection, the complete culture medium was replaced with serum- and antibiotic-free medium. Cells were incubated with transfection mixtures containing 100 nM silencing siRNA or control siRNA for 24 hours.

MicroRNA (miRNA) database searches

We utilized the miRWalk database (http://mirwalk.umm.uni-heidelberg.de/) to identify miRNAs that could bind to the MMP-2 gene. The results were further filtered using the miRDB database, resulting in the identification of 25 miRNAs that could potentially bind to the MMP-2 gene.

**Supplementary References**

1. Liu JF, Chen PC, Chang TM, Hou CH. Monocyte Chemoattractant Protein-1 promotes cancer cell migration via c-Raf/MAPK/AP-1 pathway and MMP-9 production in osteosarcoma. *Journal of experimental & clinical cancer research : CR* 2020, **39**(1)**:** 254.

2. Nguyen BT, Lin CY, Chang TK, Fong YC, Thadevoos LA, Lai CY*, et al.* Melatonin inhibits chondrosarcoma cell proliferation and metastasis by enhancing miR-520f-3p production and suppressing MMP7 expression. *Journal of pineal research* 2023**:** e12872.

3. Trang NTN, Lai CY, Tsai HC, Huang YL, Liu SC, Tsai CH*, et al.* Apelin promotes osteosarcoma metastasis by upregulating PLOD2 expression via the Hippo signaling pathway and hsa_circ_0000004/miR-1303 axis. *Int J Biol Sci* 2023, **19**(2)**:** 412-425.
