# Supplementary material for: Targeting nerve growth factor-mediated osteosarcoma metastasis: mechanistic insights and therapeutic opportunities using larotrectinib
Source: Cell Death Dis. 2024 May 30;15(5):381. doi: 10.1038/s41419-024-06752-0 (PMC11139949; doi:10.1038/s41419-024-06752-0)

Fig.2

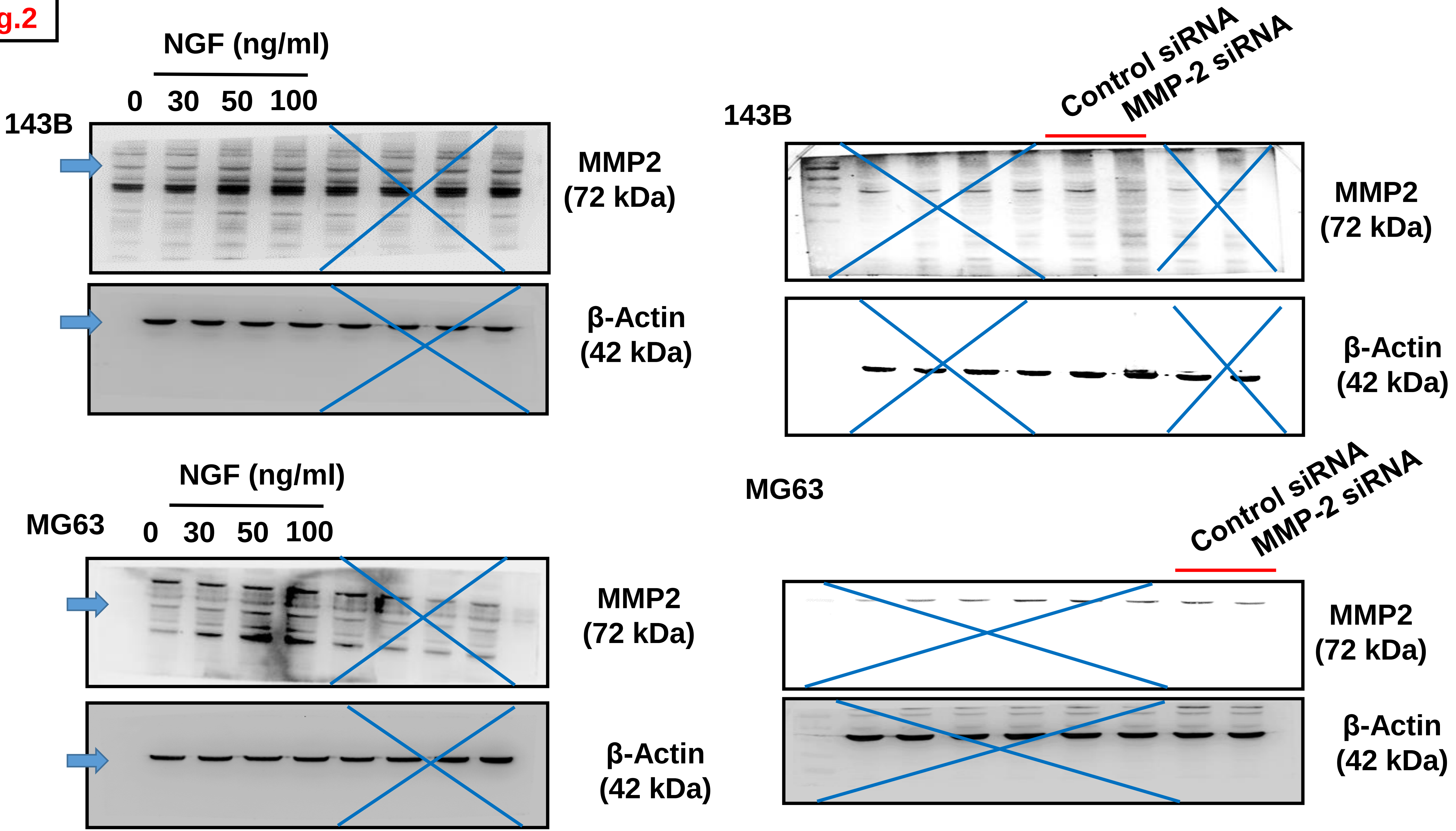

NGF (ng/ml)
0 30 50 100
143B
MMP2
(72 kDa)
β-Actin
(42 kDa)
Control siRNA
MMP-2 siRNA
143B
MMP2
(72 kDa)
β-Actin
(42 kDa)
NGF (ng/ml)
MG63
0 30 50 100
MMP2
(72 kDa)
β-Actin
(42 kDa)
MG63
Control siRNA
MMP-2 siRNA
MMP2
(72 kDa)
β-Actin
(42 kDa)

Fig.3

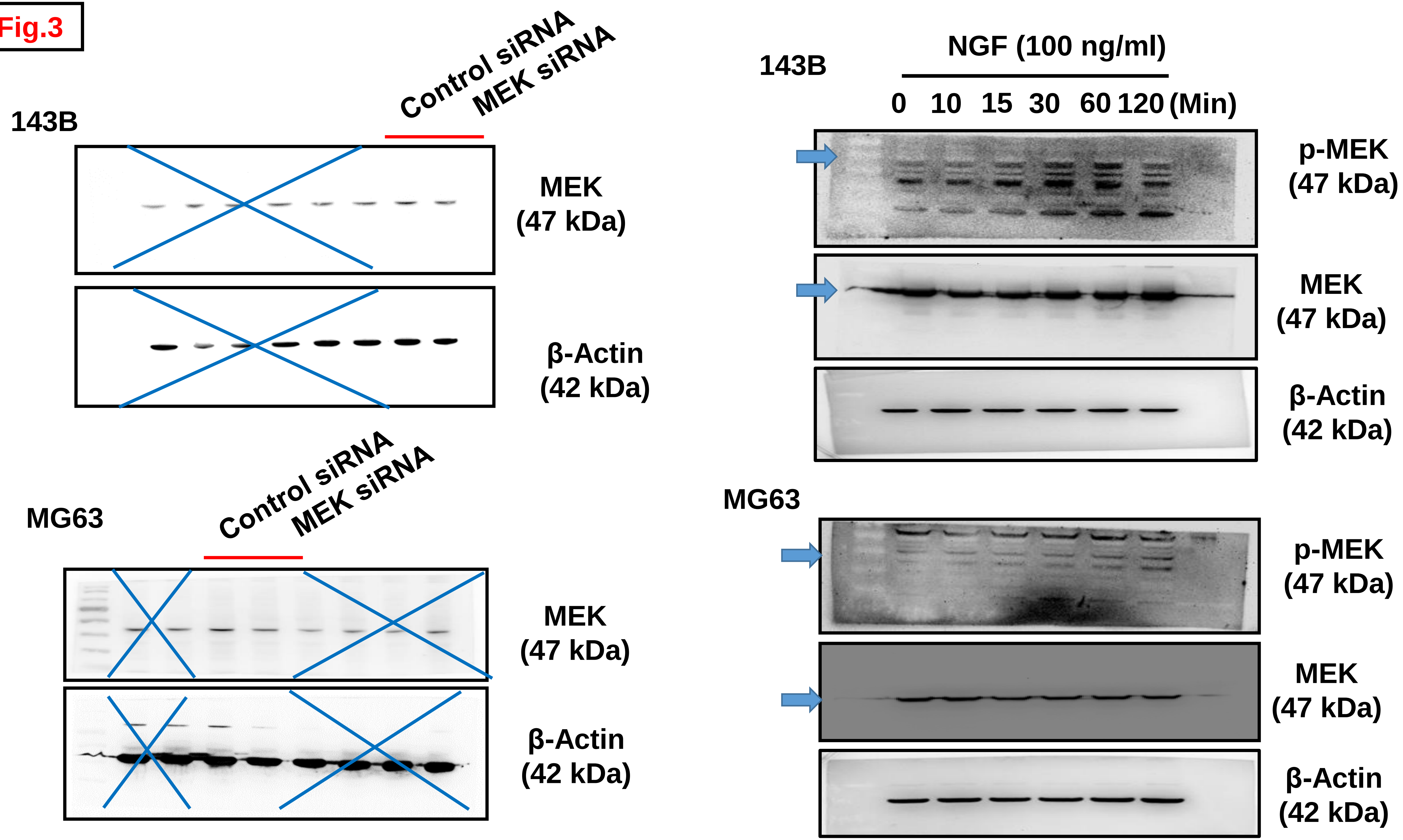
143B
Control siRNA
MEK siRNA
MEK
(47 kDa)
β-Actin
(42 kDa)
MG63
Control siRNA
MEK siRNA
MEK
(47 kDa)
β-Actin
(42 kDa)
143B
NGF (100 ng/ml)
0
10
15
30
60
120
(Min)
p-MEK
(47 kDa)
MEK
(47 kDa)
β-Actin
(42 kDa)
MG63
p-MEK
(47 kDa)
MEK
(47 kDa)
β-Actin
(42 kDa)

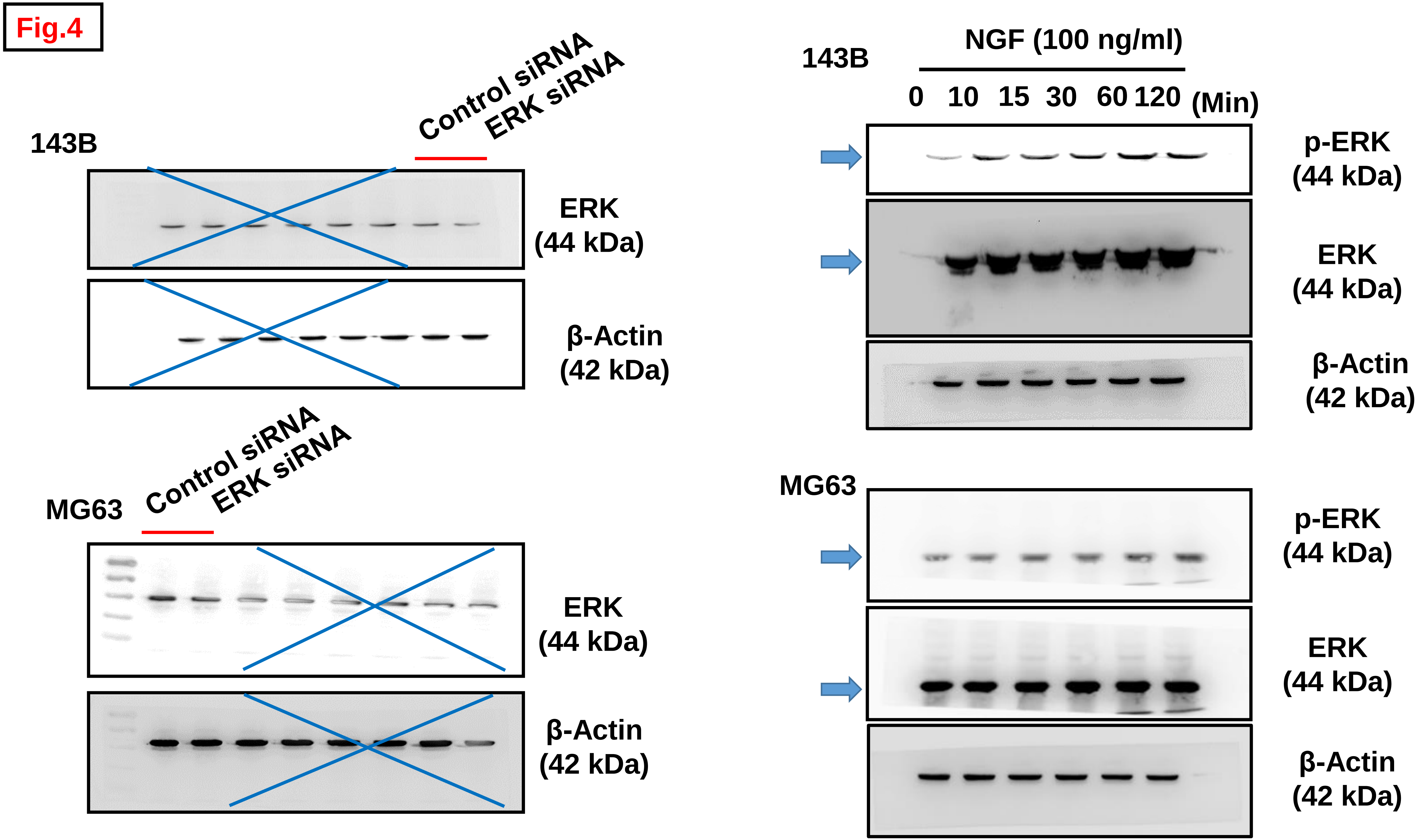
Fig.4
143B
Control siRNA
ERK siRNA
ERK
(44 kDa)
β-Actin
(42 kDa)
MG63
Control siRNA
ERK siRNA
ERK
(44 kDa)
β-Actin
(42 kDa)
143B
NGF (100 ng/ml)
0 10 15 30 60 120 (Min)
p-ERK
(44 kDa)
ERK
(44 kDa)
β-Actin
(42 kDa)
MG63
p-ERK
(44 kDa)
ERK
(44 kDa)
β-Actin
(42 kDa)

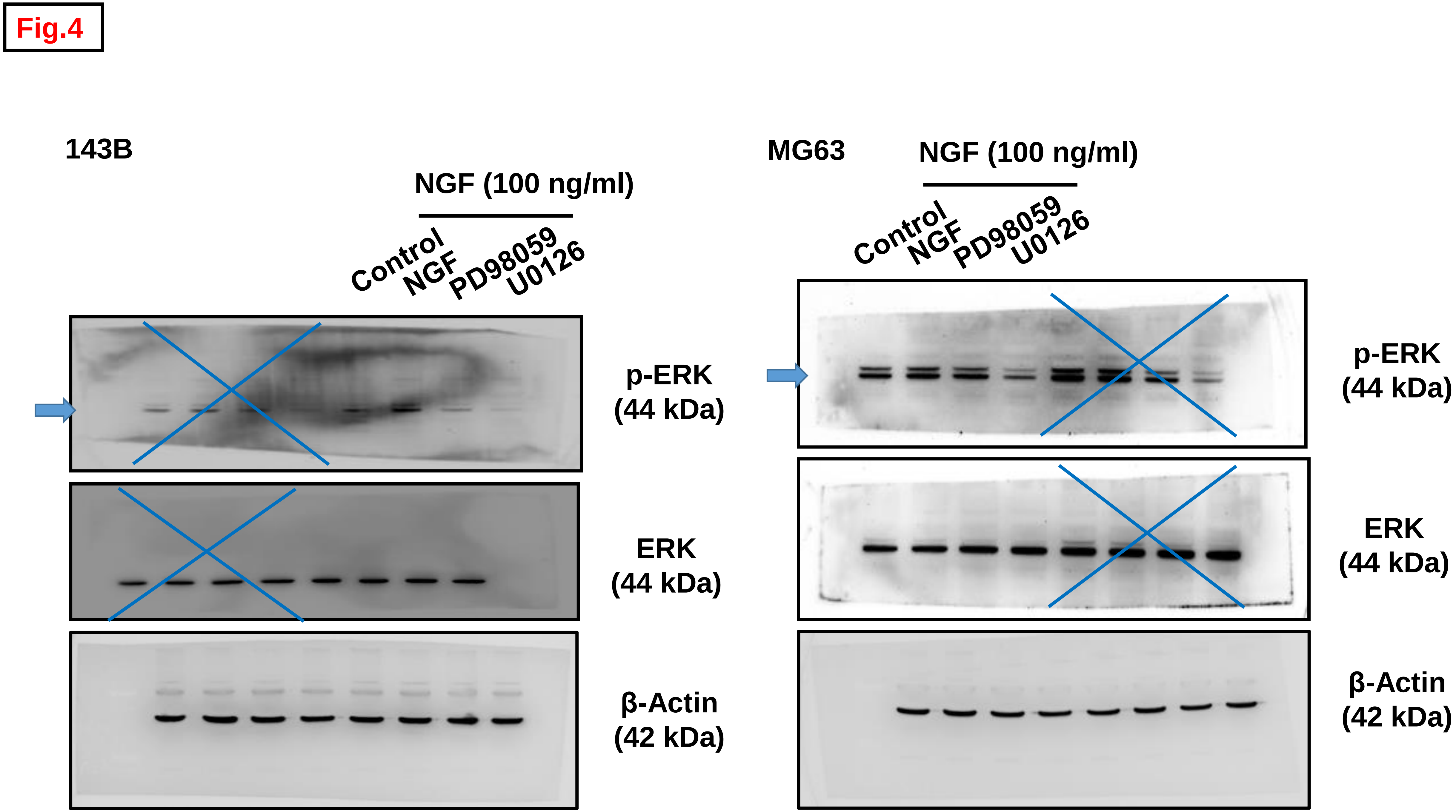
Fig.4
143B
NGF (100 ng/ml)
Control
NGF
PD98059
U0126
p-ERK
(44 kDa)
ERK
(44 kDa)
β-Actin
(42 kDa)
MG63
NGF (100 ng/ml)
Control
NGF
PD98059
U0126
p-ERK
(44 kDa)
ERK
(44 kDa)
β-Actin
(42 kDa)

Fig.5

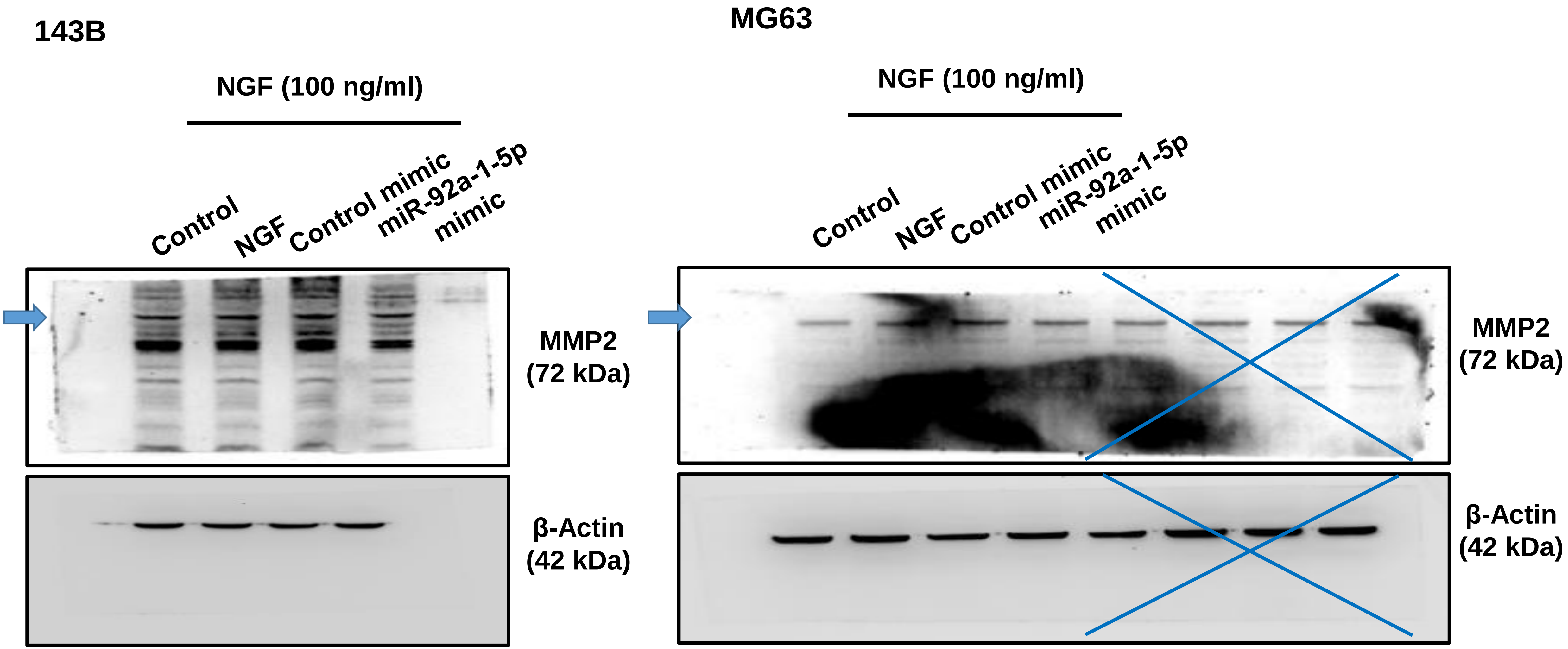

143B
NGF (100 ng/ml)
Control
NGF
Control mimic
miR-92a-1-5p mimic
MMP2 (72 kDa)
β-Actin (42 kDa)
MG63
NGF (100 ng/ml)
Control
NGF
Control mimic
miR-92a-1-5p mimic
MMP2 (72 kDa)
β-Actin (42 kDa)

Fig.6

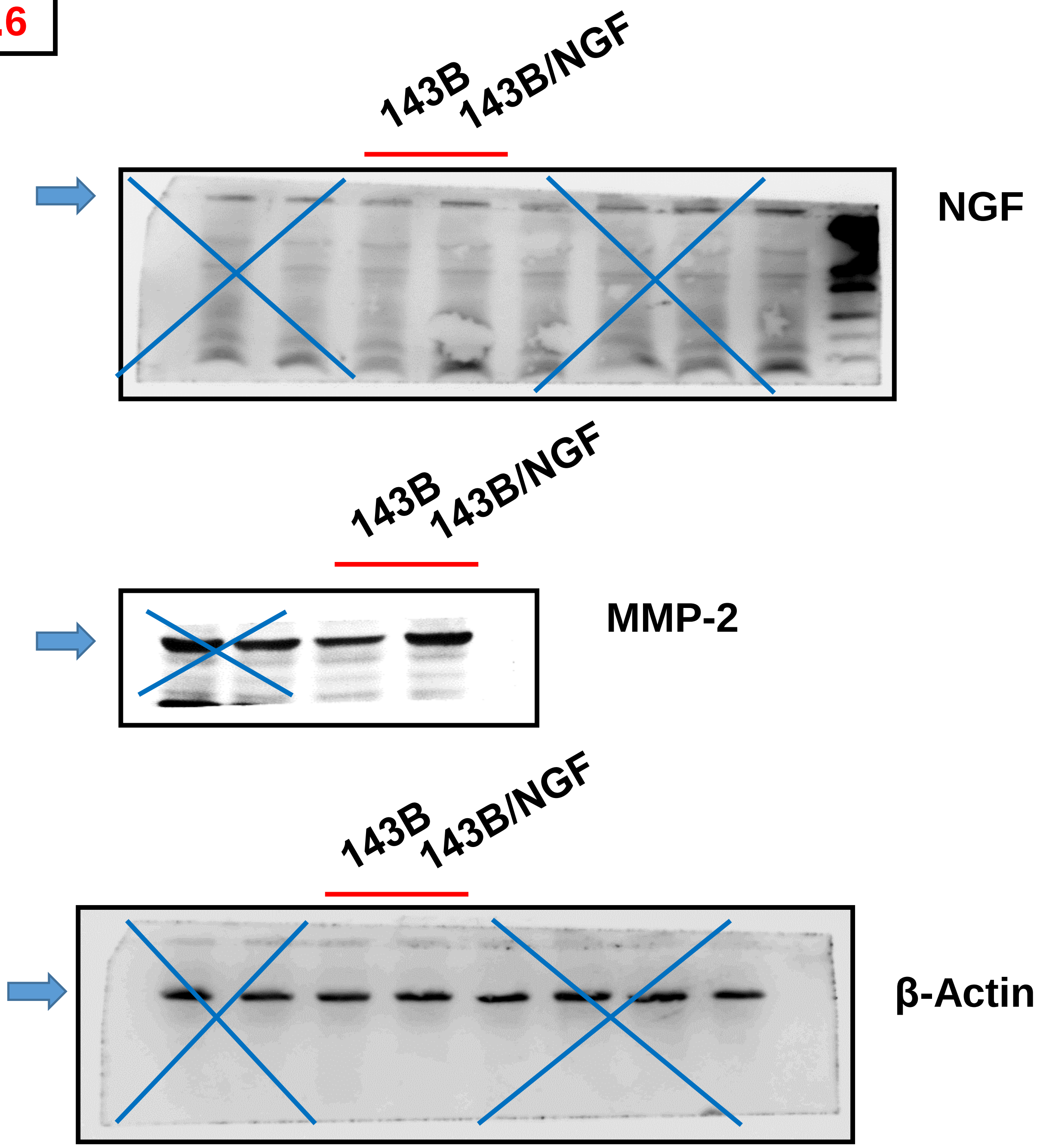

Supplement: Supplementary file 2 — Full western blots [file 41419_2024_6752_MOESM2_ESM.pdf]
